# Can Robots Earn Our Trust the Same Way Humans Do? A Systematic Exploration of Competence, Warmth, and Anthropomorphism as Determinants of Trust Development in HRI

**DOI:** 10.3389/frobt.2021.640444

**Published:** 2021-04-09

**Authors:** Lara Christoforakos, Alessio Gallucci, Tinatini Surmava-Große, Daniel Ullrich, Sarah Diefenbach

**Affiliations:** ^1^Department of Psychology, Ludwig-Maximilians-Universität München, Munich, Germany; ^2^Department of Computer Science, Ludwig-Maximilians-Universität München, Munich, Germany

**Keywords:** human-robot interaction, trust, trust development, trustworthiness, competence, warmth, anthropomorphism, social robots

## Abstract

Robots increasingly act as our social counterparts in domains such as healthcare and retail. For these human-robot interactions (HRI) to be effective, a question arises on whether we trust robots the same way we trust humans. We investigated whether the determinants competence and warmth, known to influence interpersonal trust development, influence trust development in HRI, and what role anthropomorphism plays in this interrelation. In two online studies with 2 × 2 between-subjects design, we investigated the role of robot competence (Study 1) and robot warmth (Study 2) in trust development in HRI. Each study explored the role of robot anthropomorphism in the respective interrelation. Videos showing an HRI were used for manipulations of robot competence (through varying gameplay competence) and robot anthropomorphism (through verbal and non-verbal design cues and the robot's presentation within the study introduction) in Study 1 (*n* = 155) as well as robot warmth (through varying compatibility of intentions with the human player) and robot anthropomorphism (same as Study 1) in Study 2 (*n* = 157). Results show a positive effect of robot competence (Study 1) and robot warmth (Study 2) on trust development in robots regarding anticipated trust and attributed trustworthiness. Subjective perceptions of competence (Study 1) and warmth (Study 2) mediated the interrelations in question. Considering applied manipulations, robot anthropomorphism neither moderated interrelations of robot competence and trust (Study 1) nor robot warmth and trust (Study 2). Considering subjective perceptions, perceived anthropomorphism moderated the effect of perceived competence (Study 1) and perceived warmth (Study 2) on trust on an attributional level. Overall results support the importance of robot competence and warmth for trust development in HRI and imply transferability regarding determinants of trust development in interpersonal interaction to HRI. Results indicate a possible role of perceived anthropomorphism in these interrelations and support a combined consideration of these variables in future studies. Insights deepen the understanding of key variables and their interaction in trust dynamics in HRI and suggest possibly relevant design factors to enable appropriate trust levels and a resulting desirable HRI. Methodological and conceptual limitations underline benefits of a rather robot-specific approach for future research.

## Introduction

Besides social interaction with other humans, we are increasingly confronted with innovative, intelligent technologies as our social counterparts. Social robots, which are specifically designed to interact and communicate with humans (Bartneck and Forlizzi, 2004), represent a popular example of such. They become more and more present within our everyday lives, e.g., in the field of healthcare (e.g., Beasley, 2012), but also in retail and transportation, and support us in daily tasks, like shopping or ticket purchase. Oftentimes their interaction design does not even allow a clear distinction from human counterparts, e.g., when they appear in the form of chatbots. Therefore, increasingly interacting with technology as a social counterpart in domains we have been used to cooperating with humans in, a question arises on whether we trust robots the same way we trust humans. Apart from levels of trust, this question also pertains to determinants of trust development. It thus seems worthwhile to look into theoretical foundations of trust development in interpersonal interaction, especially since trust builds a basic precondition for effective HRI (Hancock et al., 2011; van Pinxteren et al., 2019), and research in different contexts revealed a particular skepticism of machines compared to humans in trustworthiness (Dietvorst et al., 2015) and related variables such as cooperation (Merritt and McGee, 2012; Ishowo-Oloko et al., 2019), particularly relevant in consequential fields of application, such as medicine and healthcare (Promberger and Baron, 2006; Ratanawongsa et al., 2016).

In line with the general approach of transferring theories and models of interpersonal interaction to human-computer interaction (HCI) and human-robot interaction (HRI) (e.g., Gockley et al., 2006; Aly and Tapus, 2016), single studies have explored this approach with regard to trust (de Visser et al., 2016; Kulms and Kopp, 2018). Yet, they have mostly focused on single determinants and barely applied systematic manipulations of the determinants in question.

In psychological literature, a prominent conception regarding determinants of trust development is that of competence and warmth (e.g., Mayer et al., 1995; Fiske et al., 2007). The perception of both competence, i.e., an individual's capability and skills, and warmth, i.e., an individual's good intentions toward another (e.g., Mayer et al., 1995; Fiske et al., 2007), appear to foster development of trust in a human counterpart. In the context of HRI, single study results imply an according importance of similar determinants of trust development. Namely, in their metanalysis, Hancock et al. (2011) found that robot-related performance-based factors (e.g., reliability, false alarm rate, failure rate) were associated with trust development in HRI. Moreover, considering HCI in general, Kulms and Kopp (2018) have found that competence and warmth of a computer are positively related to trust development in computers.

Comparing trust in HRI to interpersonal trust, another possibly relevant determinant is anthropomorphism, namely the act of attributing human characteristics, motivations, emotions, and intentions to non-human agents (Epley et al., 2007). If we trust robots as we trust humans, the degree of a robot's human-likeness might also affect our trust in robots. Especially, since robots are increasingly being designed in an anthropomorphic way, HRI research on this determinant is currently growing. Particularly, recent studies have suggested humanlike robot design to be a promising strategy in fostering trust (e.g., Kiesler et al., 2008; Hancock et al., 2011). However, anthropomorphism has not been investigated in combination with other possible determinants to further clarify its role in trust development within HRI.

In sum, the assumingly relevant determinants of trust development in HRI, namely competence, warmth, and anthropomorphism, including their interactions, have not been comprehensively considered and systematically manipulated in HRI research. The purpose of our study was to systematically explore the transferability of determinants of interpersonal trust development (here: competence and warmth), further considering anthropomorphism as a possible influencing factor and exploring its interaction with the determinants in question. Specifically, we explored whether robot competence and warmth influence trust development in robots and what role anthropomorphism plays in this interrelation.

Results in this respect could contribute to HRI research by delivering deeper insights into conceptual relationships and underlying psychological mechanisms of trust development in HRI, shedding light on central variables and their interaction as well as examining the transferability of well-founded knowledge on interpersonal trust to HRI. Moreover, understanding what makes humans trust robots could come with implications on a societal level. It could foster a more reflected interaction with robots by highlighting reasons we trust robots in tasks such as dealing with our personal data. On a more practical level, based on the systematic manipulations of assumed relevant determinants of trust development in HRI, our research could offer insights on key design elements, which influence trust in robots and could thus be crucial in achieving desired trust levels within a particular HRI.

In the following sections we outline psychological theories and study results on determinants of interpersonal trust development, followed by recent research on determinants of trust development in HRI, reflecting on the transferability of insights. Afterwards, we present two studies each focusing on a separate combination of possible determinants of trust development in HRI and the according results and discussion. This is followed by a general discussion, considering overall limitations and future research.

## Trust Development in Interpersonal Interaction and HRI

As a multidimensional phenomenon, various definitions of trust can be found in the literature (e.g., Barber, 1983; Rempel et al., 1985; Rousseau et al., 1998). For example, in the context of technology-related trust, trust has been defined as “the attitude that an agent will help achieve an individual's goals in a situation characterized by uncertainty and vulnerability” (Lee and See, 2004, p. 54). Trust thus forms a basis for dealing with risk and uncertainty (Deutsch, 1962; Mayer et al., 1995) and fosters cooperative behavior (Corritore et al., 2003; Balliet and Van Lange, 2013). Although trust generally evolves over time and is based on multiple interactions (Rempel et al., 1985), especially in first encounters or short-time interactions, single trustee attributes may be crucial for attributed trustworthiness (e.g., Mayer et al., 2003).

### Determinants of Trust Development in Interpersonal Interaction

The broadly applied Stereotype Content Model (Fiske et al., 1999, 2002) suggests that individuals' judgment of others can be classified by the two universal dimensions of social cognition: competence and warmth. Whereas competence represents “traits that are related to perceived ability,” warmth stands for “traits that are related to perceived intent” (Fiske et al., 2007, p. 77). The authors propose that these dimensions can predict individuals' affective and behavioral responses (Fiske et al., 2007; Cuddy et al., 2008), such as the extent to which a trustor trusts the trustee. Therefore, the higher the perception of competence or warmth, the more positive the judgment, i.e., the higher the trust in the trustee.

Another model supporting the importance of these dimensions in interpersonal trust development is the widely accepted model by Mayer et al. (1995), describing trustee attributes and behaviors, such as trustworthiness, and trustor attributes, such as trust propensity, as essential determinants of trust development. Focusing on the trustee, the authors propose a three-factor model describing antecedents of trustworthiness, including ability, benevolence, and integrity. Ability represents the “group of skills, competencies, and characteristics that enable a party to have influence within some specific domain” (Mayer et al., 1995, p. 717). Benevolence represents the extent to which the trustor believes the trustee to have good intentions toward the trustor and integrity is given, when the trustor perceives that the trustee follows principles accepted by the trustor (Mayer et al., 1995). The higher these determinants are perceived, the higher the trustworthiness attributed to the trustee.

Recent study results also support the importance of similar determinants for trust development and social cognition overall. van der Werff and Buckley (2017) investigated trust development in co-worker relationships to identify cues that foster trusting behaviors. Results showed that competence and benevolence of the trustee were positively related to disclosure and reliance (van der Werff and Buckley, 2017) as forms of trust behavior (Gillespie, 2003).

Despite slightly varying terms (e.g., ability and benevolence, Mayer et al., 1995; competence and morality, Phalet and Poppe, 1997; competence and warmth, Fiske et al., 2007), competence and warmth seem to be central dimensions of individuals' perception of others. Focusing on trust, perceiving the trustee as capable of achieving certain intended goals (competence) as well as adhering to the same intentions and interests as the trustor (warmth) can foster trust development in interpersonal relationships (Mayer et al., 1995; Fiske et al., 2002, 2007).

### Transferability of Determinants of Trust Development in Interpersonal Interaction to HRI

A popular definition of trust in HRI describes trust as a “belief held by the trustor that the trustee will act in a manner that mitigates the trustor's risk in a situation, in which the trustor has put its outcomes at risk” (Wagner, 2009, p. 31). As research on trust development in HRI is relatively recent, theoretical models and studies on trust in interpersonal interaction as well as HCI can act as fundamental groundwork. Moreover, the “computers are social actors” paradigm (Nass and Moon, 2000) specifies that individuals apply social heuristics from human interactions in HCI, supporting the relevance of findings in interpersonal trust for trust in HRI. Furthermore, empirical studies show a strong correlation of trust in robots with trust in automation (Parasuraman et al., 2008; Chen et al., 2010), supporting the applicability of results regarding trust in this context to HRI (Hancock et al., 2011).

Accordingly, parallel to interpersonal trust, numerous studies have found a relevance of determinants related to robot competence for trust development in HRI. These include the robot's perceived competence based on its facial expressions (Calvo-Barajas et al., 2020), the robot's reputation in the sense of knowledge about its reliance (Bagheri and Jamieson, 2004), its previous performance (Chen et al., 2010, Lee and See, 2004), as well as its actual performance (Chen et al., 2010). Similarly, Robinette et al. (2017) found that poor robot performance was associated with a drop in self-reported trust of humans in robots, which was in turn correlated with their decision to use the robot for guidance (Robinette et al., 2017). Furthermore, in their metanalysis Hancock et al. (2011) showed that robot-related performance-based factors, such as reliability, false-alarm rate, and failure rate, predicted trust development in robots. Thus, perceiving the trustee (the robot) as competent, i.e., capable of achieving intended goals, seems essential for trust development in HRI as well.

While in HRI research warmth has not been particularly investigated as a potential determinant of trust development, assumptions can be derived from HCI literature. For example, Kulms and Kopp (2018) examined the transferability of interpersonal trust dynamics in the domain of intelligent computers, focusing on competence and warmth as possible determinants of trust in such. Competence was manipulated by means of competent (vs. incompetent) gameplay of the computer and warmth by means of unselfish (vs. selfish) game behavior of the computer. Results showed that competence and warmth were positively related to trust in computers, implying a relevance and certain transferability of trust determinants from interpersonal trust to trust in HCI.

To what degree humans actually treat technologies as social counterparts (Reeves and Nass, 1996) and apply social heuristics from human interactions (Keijsers and Bartneck, 2018) also depends on the availability of social cues, e.g., a user interface or car front looking like a smile. Thus, regarding the transferability of interpersonal trust dynamics to HRI, anthropomorphism of robots might be a relevant determinant. Accordingly, study results support a positive relationship between anthropomorphic design cues, e.g., humanlike appearance or voice of robots (Hancock et al., 2011; van Pinxteren et al., 2019) as well as agents, in general, and trust in such (e.g., Pak et al., 2012; de Visser et al., 2016, 2017). Furthermore, Kulms and Kopp (2019) explored the role of anthropomorphism and advice quality, a sort of robot competence, in trust within a cooperative human-agent setting. Results support a positive effect of anthropomorphism on self-reported trust, but also imply that competence might be essential for behavioral trust. Overall, anthropomorphism as a possible contributing factor to trust development in HRI has mainly been considered in single empirical studies in HRI research and in combination with competence in a first study on HCI (Kulms and Kopp, 2019). Such results, as well as the possibly essential role of anthropomorphism in the transferability of interpersonal trust dynamics to HRI, support a combined consideration of anthropomorphism with competence and warmth as trust determinants in HRI. Specifically, anthropomorphism may moderate the effect of competence and warmth on trust in HRI by enhancing applicability of interpersonal trust dynamics to HRI.

## Hypotheses and Research Paradigm

Based on theoretical approaches and recent findings, as summarized in the preceding paragraphs, our research explored the effect of robot competence and robot warmth on trusting a robot. We assumed that both determinants will enhance trust, focusing on two facets of trust, namely, anticipated trust toward the robot and attributed trustworthiness to the robot. We further hypothesized that this relation is mediated by individual perceptions of robot competence, which is characterized as robot warmth. In addition, we assumed that robot anthropomorphism may play a moderating role and could further strengthen the effect of robot competence and robot warmth on trust. These general hypotheses were explored in two consecutive experimental studies, each manipulating one of the possible trust determinants (Study 1: robot competence, Study 2: robot warmth). Both studies further investigated the possible moderating role of robot anthropomorphism and used the same robot and general study paradigm, consisting of experimental manipulations through a video of a specific HRI.

## Study 1

### Methods

#### Experimental Manipulation

A 2 × 2 between-subjects-design with manipulated competence (high vs. low) and manipulated anthropomorphism (high vs. low) as independent variables was applied.

For each experimental condition, a different interaction between a service robot and a human player was presented on video. In all videos the protagonists (robot and human player) were playing a shell game. The human player covered a small object with one of three shells and mixed up the shells with rapid movements. Afterwards, the robot guessed under which shell the object was hidden. Within all conditions four playthroughs were presented, all together lasting 1 min on average.

The manipulation of robot competence focused on the skills of the robot (e.g., Mayer et al., 1995; Fiske et al., 2007) in the shell game. In the condition with high competence, the robot's judgement was correct three out of four times. In the condition with low competence, the robot's judgment was correct one out of four times. Complete failure or success was avoided to allow variance within the perception of competence. To counter further possible confounding effects, e.g., of perceived warmth, the robot gave very brief answers (i.e., “left,” “right”). Finally, the total game score was illustrated on the robot's tablet after the game to support participants' notice.

Based on study results regarding explicit and implicit cues that can foster anthropomorphism (e.g., Eyssel et al., 2011; Salem et al., 2013; Waytz et al., 2014), robot anthropomorphism was manipulated explicitly through verbal (voice) and non-verbal (gestures) design cues as well as implicitly through naming the robot within the introduction given to the study. In the condition with high anthropomorphism, the robot named “Pepper” showed the shell in question with its hand and moved its head in the according direction. In the condition with low anthropomorphism, the robot did not have a name, nor did it show any gestures, or speak. Instead, its answers were presented on its tablet.

**Figure 1:**
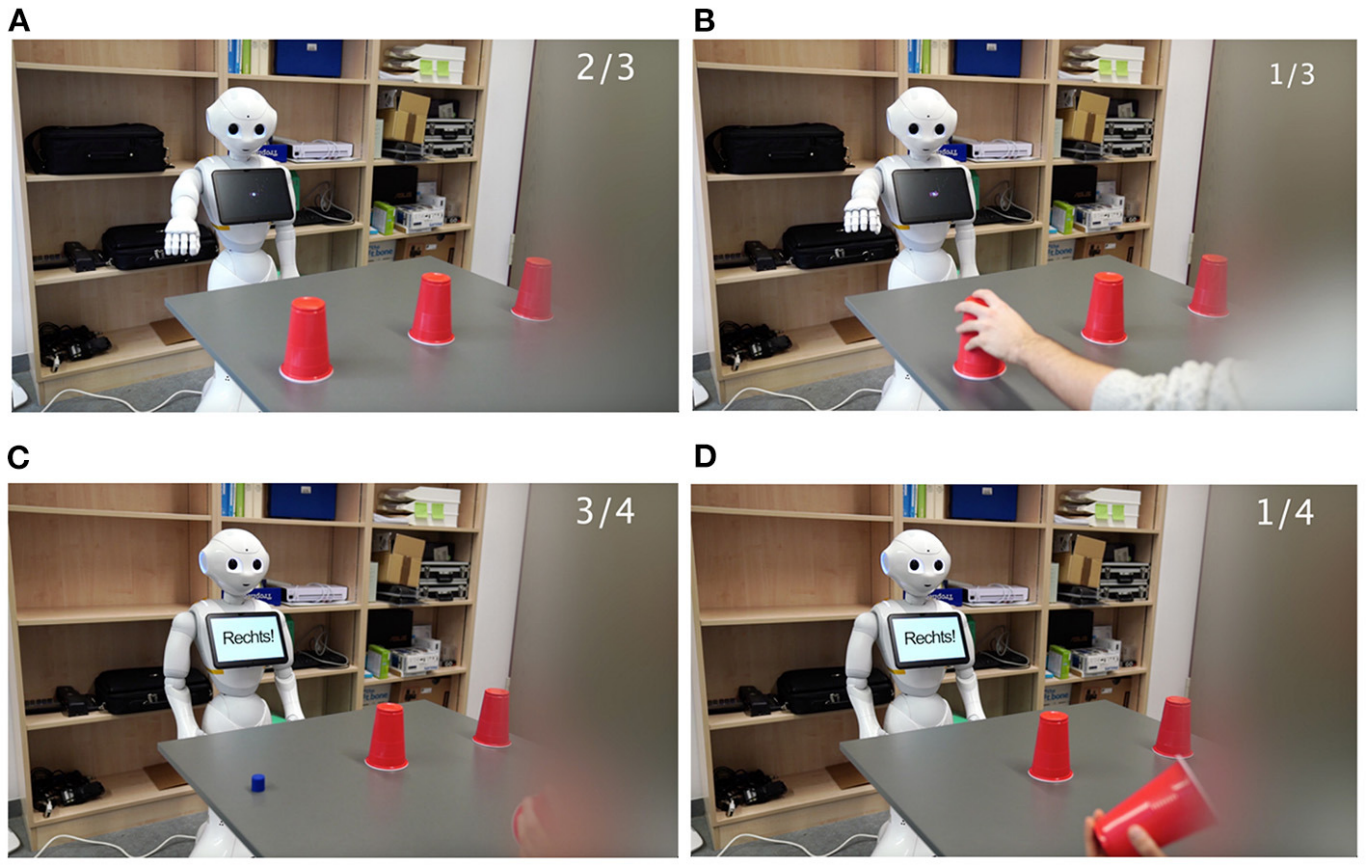
Screenshots of the videos in Study 1, displaying HRI during a shell game in the conditions **(A)** anthropomorphism high x competence high, **(B)** anthropomorphism high x competence low, **(C)** anthropomorphism low, competence high, and **(D)** anthropomorphism low, competence low. Game scores are presented in the upper right corner of each screenshot.

For the videos, the service robot Pepper by SoftBank Mobile Corp. (Pandey and Gelin, 2018) was used. According to the Wizard-of-Oz method (Fraser and Gilbert, 1991), the robot's speech and gestures were remote-controlled and triggered using the software Choreograph for Windows. Furthermore, for the robot's speech the German male voice programmed for Apple's Siri was applied. Premiere Pro, Adobe was used for overall editing. Thereby, the human player's movements, while mixing up the shells, were sped up by 50%. To avoid possible contrast effects (Bierhoff and Herner, 2002), the human counterpart in the shell game was blurred out. The four conditions are described in Table 1. In Figure 1, screenshots of the videos in all four conditions are presented.

**Table 1:** Descriptions of experimental conditions in study 1.

**Experimental conditions**	**Competence high**	**Competence low**
Anthropomorphism high	Video of shell game with robot “Pepper,” who is right in three out of four trials, speaks with a humanlike voice and points out the shell in question.	Video of shell game with robot “Pepper,” who is right in one out of four trials, speaks with a humanlike voice and points out the shell in question.
Anthropomorphism low	Video of shell game with robot, who is right in three out of four trials, presenting its answers written on its tablet's screen without voice or gestures.	Video of shell game with robot, who is right in one out of four trials, presenting its answers written on its tablet's screen without voice or gestures.

Experimental condition competence high x anthropomorphism high, n = 37.

Experimental condition competence high x anthropomorphism low, n = 41.

Experimental condition competence low x anthropomorphism high, n = 33.

*Experimental condition competence low x anthropomorphism low, n = 44*.

#### Participants

One hundred and fifty five participants between eighteen to seventy-seven years (*M* = 33.50 years, *SD* = 15.00 years; 63.87% female, 34.84% male, 1.29% diverse) took part in the study. Participants were mainly recruited via University mailing-lists and social media platforms. As an incentive for their participation, two gift coupons of thirty Euros were raffled among all participants. Alternatively, students could register their participation for course credit. There were no preconditions for participation.

#### Procedure

The study was realized via online questionnaire, using Unipark (EFS Fall 2019) for programming. The study was announced as a study on HRI. Participants were informed about the average duration of the study and available incentives. After participants informed consent regarding data privacy terms according to the German General Data Protection Regulation (DGVO) was obtained, they were randomly assigned to one of four experimental conditions. In each condition participants watched the video of the above-described HRI and afterwards provided different judgements on the robot and additional measures as further specified below. All measures were assessed in German, using pre-tested translations if no validated versions were available.

#### Measures

##### Anticipated Trust

Anticipated trust toward the robot as one measure of trust in our study was measured by the five-item Faith subscale of the measure for human-computer trust by Madsen and Gregor (2000) (e.g., If I am not sure about a decision, I have faith that the system will provide the best solution). Items were assessed on a seven-point Likert-Scale (1 = “does not apply at all”; 7 = “applies fully”) and showed an internal consistency of α = 0.88.

##### Attributed Trustworthiness

Attributed trustworthiness to the robot as the second measure of trust in our study was measured by a six-item scale of terms for assessing trustworthiness as a dimension of credibility of computer products by Fogg and Tseng (1999). The item “well-intentioned” was excluded to minimize confounding effects with robot warmth. The resulting five items (i.e., trustworthy, good, truthful, unbiased, honest) were assessed on a five-point Likert-Scale (1 = “does not apply at all”; 5 = “applies fully”) and showed an internal consistency of α = 0.79.

##### Perceived Anthropomorphism

Participants' perceived anthropomorphism of the robot was measured by a single item (i.e., The robot made a humanlike impression), assessed on a five-point Likert Scale (1 = “does not apply at all”; 5 = “applies fully”).

##### Perceived Competence

Participants' perceived competence of the robot was measured by means of the six-item Competence scale by Fiske et al. (2002), initially developed to assess stereotypes in interpersonal interaction. Items (i.e., competent, confident, capable, efficient, intelligent, skilful) were assessed on a seven-point Likert Scale (1 = “does not apply at all”; 7 = “applies fully”) and showed an internal consistency of α = 0.84.

##### Perceived Warmth

Participants' perceived warmth of the robot was measured by means of the six-item Warmth scale by Fiske et al. (2002), initially developed to assess stereotypes in interpersonal interaction. The item “trustworthy” was excluded to minimize confounding effects with attributed trustworthiness. The resulting five items (i.e., friendly, well-intentioned, warm, good-natured, sincere) were assessed on a seven-point Likert Scale (1 = “does not apply at all”; 7 = “applies fully”) and showed an internal consistency of α = 0.93.

##### Individual Tendency to Anthropomorphize

Participants' individual tendency to anthropomorphize was measured by means of the ten-item AQcurrent subscale of the Anthropomorphism Questionnaire by Neave et al. (2015). Items (e.g., I sometimes wonder if my computer deliberately runs more slowly after I shouted at it) were assessed on a seven-point Likert Scale (1 = “does not apply at all”; 7 = “applies fully”) and showed an internal consistency of α = 0.86.

##### Experience With Technology/Robots

Participants' experience with technology and robots were each measured by a self-constructed item (i.e., I generally consider my knowledge and skills in the field of technology/robots to be high). Items were assessed on a five-point Likert Scale (1 = “does not apply at all”; 5 = “applies fully”).

##### Attitude Toward Robots

Participants' attitude toward robots was measured by means of the four-item Attitude Toward Robots subscale of the Robot Acceptance Questionnaire by Wu et al. (2014). Items (e.g., The robot would make life more interesting and stimulating in the future) were assessed on a five-point Likert Scale (1 = “does not apply at all”; 5 = “applies fully”) and showed an internal consistency of α = 0.90.

##### Demographic Measures

Participant's age was assessed by means of an open question. Gender was assessed through a single choice question with three answer options (i.e., male, female, diverse).

#### Hypotheses

Based on the above derived general hypotheses we specified the following for Study 1.

H1a: Individuals confronted with the robot with high competence (vs. low competence) will show higher anticipated trust.
H1b: Individuals confronted with the robot with high competence (vs. low competence) will attribute higher trustworthiness to the robot.
H2a: The effect of manipulated competence on anticipated trust is mediated through perceived competence of the robot.
H2b: The effect of manipulated competence on attributed trustworthiness is mediated through perceived competence of the robot.
H3a: The effect of manipulated competence on anticipated trust is strengthened by manipulated anthropomorphism.
H3b: The effect of manipulated competence on attributed trustworthiness is strengthened by manipulated anthropomorphism.

### Results

Analyses were conducted with SPSS (IBM Statistics Version 26). For mediation and moderation analyses the Process Macro (Hayes and Preacher, 2013) was used.

#### Preliminary Analyses

Means, standard deviations, and Pearson correlations of the variables within the overall sample of Study 1 are illustrated in Table 2.

**Table 2:** Means (*M*), standard deviations (*SD*), and Pearson correlations of relevant variables within the overall sample of study 1.

**Variable**	***M***	***SD***	**1**	**2**	**3**	**4**	**5**	**6**	**7**	**8**	**9**	**10**
1. Age	33.5	15.00	–									
2. Anticipated trust	2.57	1.23	0.09	–								
3. Trustworthiness	2.97	0.86	−0.06	0.40^**^	–							
4. Perceived competence	3.55	1.34	−0.15	0.41^**^	0.69^**^	–						
5. Perceived anthropomorphism	2.22	1.11	−0.06	0.14	0.41^**^	0.25^**^	–					
6. Perceived warmth	3.45	1.53	−0.29^**^	0.14	0.46^**^	0.44^**^	0.39^**^	–				
7. Individual tendency to anthropomorphize	2.36	1.15	−0.27^**^	0.15	0.14	0.29^**^	0.11	0.27^**^	–			
8. Experience with technology	4.01	1.69	0.05	0.09	0.15	0.04	0.17^*^	0.17^*^	−0.07	-		
9. Experience with robots	2.61	1.68	0.08	0.16^*^	0.15	0.08	0.14	0.10	−0.02	0.73^**^	–	
10. Attitude toward robots	4.31	1.52	−0.08	0.16^*^	0.34^**^	0.27^**^	0.19^*^	0.31^**^	0.14	0.31^**^	0.25^**^	–

* Indicates p < 0.05.

** *Indicates p < 0.01*.

One-way ANOVAs showed no effect of the experimental conditions on age [*F*_(3,151)_ = 0.69, *p* = 0.562, η^2^ = 0.013], individual tendency to anthropomorphize [*F*_(3,151)_ = 0.39, *p* = 0.763, η^2^ = 0.008], experience with technology [*F*_(3,151)_ = 0.50, *p* = 0.687, η^2^ = 0.010], experience with robots [*F*_(3,151)_ = 1.01, *p* = 0.354, η^2^ = 0.021], or attitude toward robots [*F*_(3,151)_ = 1.65, *p* = 0.180, η^2^ = 0.032]. The conducted Pearson's chi-squared test showed that experimental conditions did not differ significantly in gender distribution *X*^2^ (6, *N* = 155) = 4.19, *p* = 0.651). Thus, there were no systematic differences regarding these variables to be further considered.

Furthermore, conducted one-way ANOVAs for manipulation checks showed that, as intended, manipulated competence had a significant effect on perceived competence [*F*_(1,153)_ = 44.47, *p* < 0.001, η^2^_*p*_ = 0.225] as mean perceived competence was higher for conditions of high competence (*M* = 4.18, *SD* = 1.26) than low competence (*M* = 2.90, *SD* = 1.12). Additionally, according to our manipulation, manipulated anthropomorphism had a significant effect on perceived anthropomorphism [*F*_(1,153)_ = 12.81, *p* < 0.001, η^2^_*p*_ = 0.077] as mean perceived anthropomorphism was higher for conditions of high anthropomorphism (*M* = 2.56, *SD* = 1.16) than low anthropomorphism (*M* = 1.94, *SD* = 0.98).

#### Hypotheses Testing

Two separate two-way ANOVAs were conducted to test the assumed effects of competence and anthropomorphism on anticipated trust (H1a, H3a) and attributed trustworthiness (H1b, H3b).

Regarding anticipated trust, the conducted two-way ANOVA showed a significant effect of manipulated competence [*F*_(3,151)_ = 25.64, *p* < 0.001, η^2^_*p*_ = 0.145] but not manipulated anthropomorphism [*F*_(3,151)_ = 0.24, *p* = 0.602, η^2^_*p*_ = 0.002]. No interaction effect of manipulated competence and manipulated anthropomorphism on anticipated trust [*F*_(3,151)_ = 0.681, *p* = 0.411, η^2^_*p*_ = 0.004] was found. Mean anticipated trust was higher for conditions of high competence (*M* = 3.03; *SD* = 1.11) compared to low competence (*M* = 2.10; *SD* = 1.17). Thus, H1a was supported. No moderation effect of manipulated anthropomorphism on the effect of manipulated competence on anticipated trust was found. Thus, H3a was not supported.

Regarding attributed trustworthiness, the conducted two-way ANOVA showed a significant effect of manipulated competence [*F*_(3,151)_ = 17.01, *p* < 0.001, η^2^_*p*_ = 0.102] but not manipulated anthropomorphism [*F*_(3,151)_ = 3.02, *p* = 0.085, η^2^_*p*_ = 0.020]. No interaction effect of manipulated competence and manipulated anthropomorphism on attributed trustworthiness [*F*_(3,151)_ = 2.06, *p* = 0.153, η^2^_*p*_ = 0.013] was found. Mean attributed trustworthiness was higher for conditions of high competence (*M* = 3.23; *SD* = 0.80) compared to low competence (*M* = 2.70; *SD* = 0.83). Thus, H1a was supported. No moderation effect of manipulated anthropomorphism on the effect of manipulated competence on attributed trustworthiness was found. Thus, H3a was not supported.

The conducted mediated regression analysis showed a positive total effect of manipulated competence on anticipated trust (*B* = 0.93, *t* = 5.05, *p* < 0.001) and that perceived competence significantly mediated this interrelation with a positive indirect effect (*B* = 0.35). A bootstrap 95% CI around the indirect effect did not contain zero (0.14; 0.61). The direct effect of manipulated competence on anticipated trust remained significant (*B* = 0.58, *t* = 2.87, *p* = 0.005) after including the mediator variable, implying a partial mediation, and partially supporting H2a. A detailed overview of the mediated regression analysis is presented in Table 3.

**Table 3:** Mediated regression analysis testing the effect of manipulated competence on anticipated trust mediated by perceived competence within study 1.

					**Model**
**Predictor**	***B***	***SE***	***t***	***P***	***R^**2**^***
Model 1: X on Y					0.14
Intercept	2.10	0.13	16.13	<0.001	
Manipulated competence	0.93	0.18	5.05	<0.001	
Model 2: X on M					0.23
Intercept	2.90	0.14	21.42	<0.001	
Manipulated competence	1.27	0.19	6.67	<0.001	
Model 3: X + M on Y					0.21
Intercept	1.30	0.25	5.19	<0.001	
Perceieved competence	0.28	0.08	3.70	<0.001	
Manipulated competence	0.58	0.20	2.87	0.005	

The conducted mediated regression analysis showed a positive total effect of manipulated competence on attributed trustworthiness (*B* = 0.53, *t* = 4.05; *p* < 0.001) and that perceived competence significantly mediated this interrelation with a positive indirect effect (*B* = 0.56). A bootstrap 95% CI around the indirect effect did not contain zero (0.37; 0.78). The direct effect of manipulated competence on attributed trustworthiness became not significant (*B* = −0.03, *t* = −0.28, *p* = 0.784) after including the mediator variable, implying a complete mediation, and supporting H2b. A detailed overview of the mediated regression analysis is presented in Table 4.

**Table 4:** Mediated regression analysis testing the effect of manipulated competence on attributed trustworthiness mediated by perceived competence within study 1.

					**Model**
**Predictor**	***B***	***SE***	***T***	***P***	***R^**2**^***
Model 1: X on Y					0.10
Intercept	2.70	0.09	28.98	<0.001	
Manipulated competence	0.53	0.13	4.05	<0.001	
Model 2: X on M					0.23
Intercept	2.90	0.14	21.42	<0.001	
Manipulated competence	1.27	0.19	6.67	<0.001	
Model 3: X + M on Y					0.47
Intercept	1.41	0.14	9.90	<0.001	
Perceived competence	0.44	0.04	10.37	<0.001	
Manipulated competence	−0.03	0.11	−0.27	0.784	

#### Exploratory Analyses

Exploratory analyses were performed to detect possible interrelations between the studied constructs beyond our predefined hypotheses. Hence, we tested effects of manipulated competence on perceived anthropomorphism as well as effects of manipulated anthropomorphism on perceived competence. Two one-way ANOVAs showed no effect of manipulated competence on perceived anthropomorphism [*F*_(1,153)_ = 0.55, *p* = 0.460; η^2^_*p*_ = 0.004] but a significant effect of manipulated anthropomorphism on perceived competence [*F*_(1,153)_ = 4.28, *p* = 0.040; η^2^_*p*_= 0.027]. Thereby, mean perceived competence was higher for conditions of high anthropomorphism (*M* = 3.79; *SD* = 1.38) compared to low anthropomorphism (*M* = 3.35; *SD* = 1.29).

Furthermore, we conducted moderation analyses in parallel to the assumed interaction effect between competence and anthropomorphism on trust (H3), however, this time considering the participants' subjective perceptions of robot competence and robot anthropomorphism instead of the experimental factors as predictors of trust. Regarding anticipated trust as one trust measure, only perceived competence showed as a significant predictor (*B* = 0.38, *t* = 2.57, *p* = 0.011), whereas perceived anthropomorphism (*B* = 0.06, *t* = 0.25, *p* = 0.806) and the interaction of perceived competence and perceived anthropomorphism (*B* = −0.00, *t* = −0.07, *p* = 0.945) did not. Perceived anthropomorphism therefore did not moderate the effect of perceived competence on anticipated trust. Regarding attributed trustworthiness as the other trust measure, perceived competence (*B* = 0.53, *t* = 6.96; *p* < 0.001), perceived anthropomorphism (*B* = 0.42, *t* = 3.55; *p* < 0.001), as well as the interaction of perceived competence and perceived anthropomorphism (*B* = −0.06, *t* = −2.00, *p* = 0.047), showed as significant predictors. Perceived anthropomorphism therefore moderated the effect of perceived competence on attributed trustworthiness. A detailed overview of the moderation analysis is presented in Table 5.

**Table 5:** Moderated regression analysis testing the effect of perceived competence on attributed trustworthiness moderated by perceived anthropomorphism within study 1.

					**Model**
**Predictor**	***B***	***SE***	***T***	***P***	***R^**2**^***
Model					0.54
Intercept	0.65	0.28	2.33	0.021	
Perceived competence	0.53	0.08	6.96	<0.001	
Perceived anthropomorphism	0.42	0.12	3.55	<0.001	
Perceived competence ^*^ perceived anthropomorphism	−0.06	0.03	−2.00	0.047	

* *stand for interaction*.

### Discussion

The aim of Study 1 was to investigate the influence of robot competence on trust in HRI as well as the role of robot anthropomorphism in this interrelation. In this regard we manipulated robot competence and robot anthropomorphism in videos, in which a robot played a shell game with a human player. Based on the robot's behavior in this HRI, study participants provided two types of trust ratings, namely, anticipated trust toward the robot and attributed trustworthiness to the robot. In conformity with our hypotheses, manipulated competence had a significant positive effect on anticipated trust as well as attributed trustworthiness and both interrelations were (partially) mediated by perceived competence. Thus, according to our findings, robot competence appears to be a possible determinant of trust development in HRI, supporting the transferability of competence as a determinant of trust development in interpersonal interaction (e.g., Mayer et al., 1995; Fiske et al., 2007) to HRI. In addition, our results are compatible with previous HRI research (e.g., Hancock et al., 2011; Robinette et al., 2017), implying a positive effect of robot competence on trust in robots.

However, contrary to our hypotheses, manipulated anthropomorphism did not moderate the effect of manipulated competence on the trust ratings. This might be rooted in a rather restricted variance of anthropomorphism due to the manipulation based on the same robot, with the identical visual appearance in both conditions. Previous results that revealed an effect of anthropomorphic agent design have used stronger manipulations, e.g., comparing different types of agents, such as computers vs. avatars (e.g., de Visser et al., 2016). Yet, exploratory analyses revealed that the perception of the robot as anthropomorphic may still play a role, given that the individually perceived anthropomorphism (as well as perceived competence) predicted trust in the robot. In addition, the individually perceived anthropomorphism moderated the effect of perceived competence on attributed trustworthiness. In sum, this underlines the role of individual perception for the formation of psychological judgments such as trust and hints at a further consideration of robot anthropomorphism as a determinant of trust development in HRI, especially in combination with other known relevant determinants, such as competence. This finding can be considered in line with study results, showing that humans lose confidence in erring computers quicker than erring humans, highlighting the role of competence for trust in HCI as well as indicating a possible interaction of competence and anthropomorphism in this regard (Dietvorst et al., 2015). Similarly, previous results by de Visser et al. (2016) found that an increasing (feedback) uncertainty regarding a robot's performance during a task magnified the effect of agent anthropomorphism on trust resilience, i.e., a higher resistance to breakdowns in trust. The authors argue that “increasing anthropomorphism may create a protective resistance against future errors” (de Visser et al., 2016), indicating an interaction of robot competence and robot anthropomorphism. Our second study explored warmth as a further potential determinant of trust, again in combination with anthropomorphism.

## Study 2

### Methods

#### Experimental Manipulation

A 2 × 2 between-subjects-design with manipulated warmth (high vs. low) and manipulated anthropomorphism (high vs. low) as independent variables was applied.

For each experimental condition, a different interaction between a service robot and a human player was presented on video. In all videos the protagonists (a robot and two human players) were playing a shell game. This time, human player 1 covered a small object with one of three shells and mixed up the shells with rapid movements. Afterwards, human player 2 guessed under which shell the object was hidden. The robot was standing next to human player 2 and appearing to also observe the game. Within all conditions three playthroughs were presented, all together lasting 1 min on average. In the first playthrough human player 2 guesses wrongly without consulting the robot, in the two following playthroughs human player 2 expresses a guess and the robot additionally consults afterwards.

The manipulation of robot warmth focused on the intentions of the robot (Mayer et al., 1995; Fiske et al., 2007) regarding the shell game. In the condition with high warmth, the robot had the same intentions and interests as human player 2 (human player 2 winning at the shell game). This was expressed by the robot showing compassion after the first lost playthrough and offering help. In the following playthroughs the robot consults human player 2 correctly and cheers after each win. In the condition with low warmth, the robot had opposed intentions and interests to human player 2 (human player 2 losing at the shell game). This was expressed by the robot depreciating human player 2 after the first lost playthrough, yet offering help. Human player 2 accepts the robot's help but loses at the second playthrough because of the robot's misleading advice. The robot cheers gleefully. In the third playthrough the robot again advises human player 2 on the decision. Yet, human player 2 does not follow the robot's advice and decides correctly, which the robot gets miffed at. To counter further possible confounding effects, e.g., of perceived competence, the robot appeared to know the correct answer in both conditions, as a basis to help (warmth high) or mislead (warmth low) human player 2. In addition, human player 2 always expressed an assumption before consulting the robot. Robot anthropomorphism was again manipulated explicitly through verbal (voice) and non-verbal (gestures) design cues as well as implicitly through naming the robot within the introduction given to the study. In the condition anthropomorphism high, the robot named “Pepper” verbally expressed its advice. Furthermore, it turned its head in the direction of player 2 while speaking. In the condition with low anthropomorphism, the robot did not have a name, nor did it show any gestures or speak. Instead, its advice was presented on its tablet.

**Figure 2:**
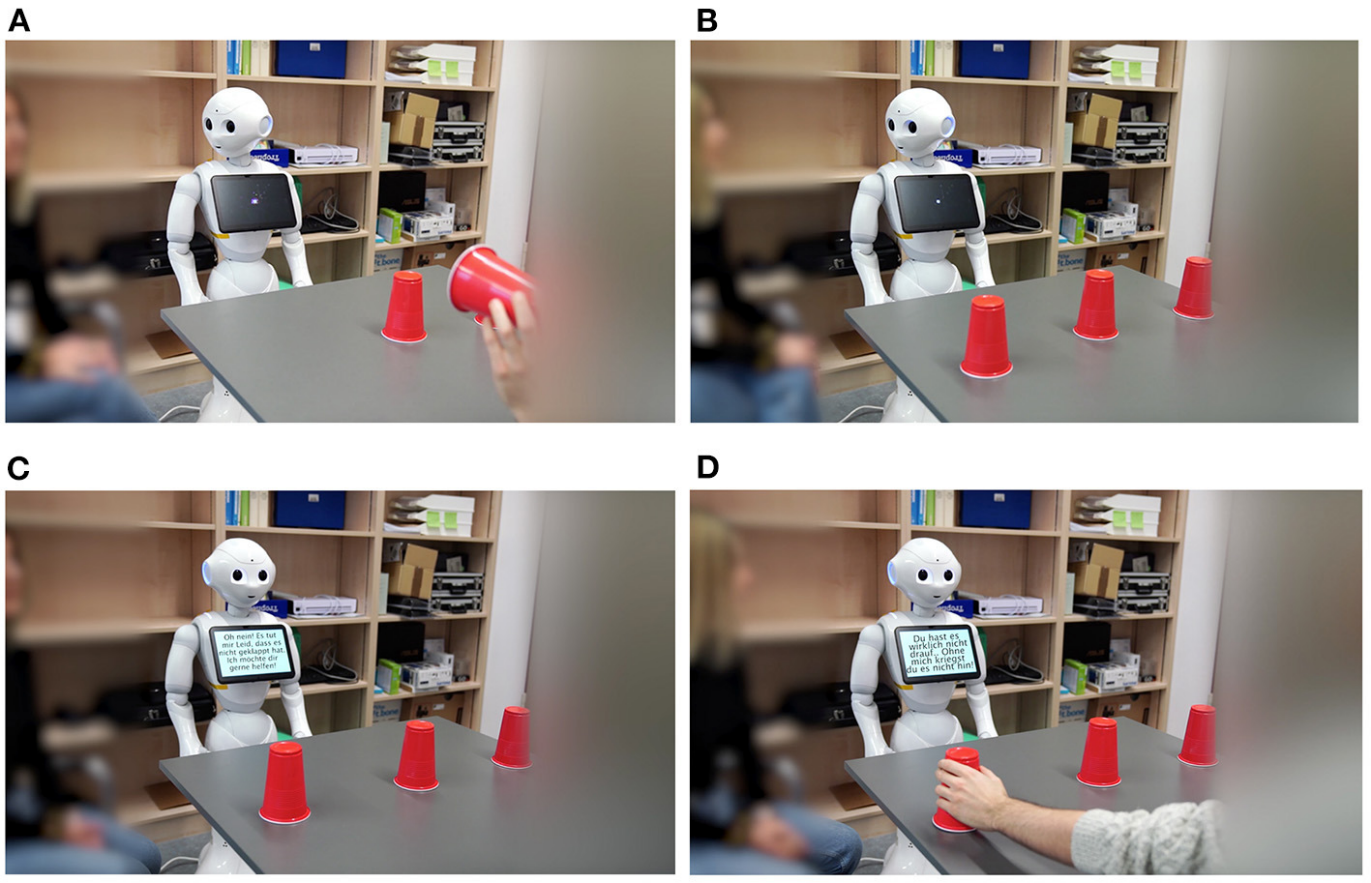
Screenshots of the videos in Study 2, displaying HRI during a shell game in the conditions **(A)** anthropomorphism high x warmth high, **(B)** anthropomorphism high x warmth low, **(C)** anthropomorphism low warmth high, and **(D)** anthropomorphism low warmth low.

For the videos, the same service robot as in Study 1 was used and the same method, software, and voice were used for the robot's speech and gestures. Similarly, the same program as in Study 1 was used for overall editing. In Study 2, human player 1's movements were not sped up, to not make guessing correctly appear highly competent in itself and cause possible confounding effects. Again, the human counterparts in the shell game were blurred out. The four conditions are described in Table 6. In Figure 2 screenshots of the videos in all four conditions are presented.

**Table 6:** Descriptions of experimental conditions in study 2.

**Experimental conditions**	**Warmth high**	**Warmth low**
High anthropomorphism	Video of shell game with robot “Pepper” consulting player 2 according to the player's interest, speaking with a humanlike voice and turning its head toward player 2 while speaking.	Video of shell game with robot “Pepper” consulting player 2 against the player's interest, speaking with a humanlike voice and turning its head toward player 2 while speaking.
Low anthropomorphism	Video of shell game with robot consulting player 2 according to the player's interest, presenting its advice written on its tablet's screen without voice or gestures.	Video of shell game with robot consulting player 2 against the player's interest, presenting its advice written on its tablet's screen without voice or gestures.

*Experimental condition warmth high x anthropomorphism high, n = 40; Experimental condition warmth high x anthropomorphism low, n = 37; Experimental condition warmth low x anthropomorphism high, n = 39; Experimental condition warmth low x anthropomorphism low, n = 41*.

#### Participants

One hundred and fifty seven participants between eighteen to sixty-seven years (*M* = 34.53 years, *SD* = 13.88 years; 60.51% female, 39.49% male) took part in the study. Participant recruiting method and offered incentives were the same as in Study 1. Again, there were no preconditions for participation.

#### Procedure

The study procedure was the exact same as in Study 1, except one detail regarding the order of measures in the survey. Namely, perceived warmth was assessed before perceived competence.

#### Measures

The applied measures were the same as in Study 1. All scales showed satisfactory internal scale consistency (anticipated trust: α = 0.88, attributed trustworthiness: α = 0.88, perceived warmth: α = 0.94, perceived competence: α = 0.84, individual tendency to anthropomorphize: α = 0.83, attitude toward robots: α = 0.91).

#### Hypotheses

Based on the above derived general hypotheses we specified the following for Study 2.

H1a: Individuals confronted with the HRI with the robot with high warmth (vs. low warmth) will show higher anticipated trust.
H1b: Individuals confronted with the HRI with the robot with high warmth (vs. low warmth) will attribute higher trustworthiness to the robot.
H2a: The effect of manipulated warmth on anticipated trust is mediated through perceived warmth of the robot.
H2b: The effect of manipulated warmth on attributed trustworthiness is mediated through perceived warmth of the robot.
H3a: The effect of manipulated warmth on anticipated trust is strengthened by manipulated anthropomorphism.
H3b: The effect of manipulated warmth on attributed trustworthiness is strengthened by manipulated anthropomorphism.

### Results

Analyses were conducted with SPSS (IBM Statistics Version 26). For mediation and moderation analyses the Process Macro (Hayes and Preacher, 2013) was used.

#### Preliminary Analyses

Means, standard deviations, and Pearson correlations of the variables within the overall sample of Study 2 are illustrated in Table 7.

**Table 7:** Means (*M*), standard deviations (*SD*), and Pearson correlations of relevant variables within the overall sample of study 2.

**Variable**	***M***	***SD***	**1**	**2**	**3**	**4**	**5**	**6**	**7**	**8**	**9**	**10**
1. Age	34.53	13.88	–									
2. Anticipated trust	3.14	1.43	0.16^*^	–								
3. Trustworthiness	2.78	1.07	0.09	0.45^**^	–							
4. Perceived warmth	3.55	1.75	0.12	0.33^**^	0.74^**^	–						
5. Perceived anthropomorphism	2.44	1.19	−0.05	0.14	0.27^**^	0.27^**^	–					
6. Perceived competence	4.08	1.38	−0.09	0.48^**^	0.49^**^	0.41^**^	0.32^**^	–				
7. Individual tendency to anthropomorphize	2.21	1.01	−0.10	0.17^*^	−0.02	0.02	0.21^**^	0.15	–			
8. Experience with technology	4.40	1.71	0.00	−0.03	0.02	0.04	−0.02	−0.12	0.03	–		
9. Experience with robots	2.82	1.67	0.06	0.15	0.11	0.09	0.02	−0.06	0.03	0.61^**^	–	
10. Attitude toward robots	4.10	1.60	0.17^*^	0.17^*^	0.08	0.11	0.05	0.05	0.04	0.26^**^	0.32^**^	–

* Indicates p < 0.05,

** *Indicates p < 0.01*.

One-way ANOVAs showed no effect of the experimental conditions on age [*F*_(3,153)_ = 0.92, *p* = 0.431, η^2^_*p*_ = 0.018], individual tendency to anthropomorphize [*F*_(3,153)_ = 1.71, *p* = 0.168, η^2^_*p*_ = 0.032], experience with robots [*F*_(3,153)_ = 0.65, *p* = 0.568, η^2^_*p*_ = 0.013], experience with technology [*F*_(3,153)_ = 0.70, *p* = 0.557, η^2^_*p*_ = 0.013], or attitude toward robots [*F*_(3,153)_ = 1.18, *p* = 0.320, η^2^_*p*_ = 0.023]. The conducted Pearson's chi-squared test showed that experimental conditions did not differ significantly in gender distribution [*X*^2^_(3,N = 157)_ =1.79, *p* = 0.617]. Thus, there were no systematic differences regarding these variables to be further considered.

Furthermore, conducted one-way ANOVAs for manipulation checks showed that, as intended, manipulated warmth had a significant effect on perceived warmth [*F*_(1,155)_ = 62.63, *p* < 0.001, η^2^_*p*_= 0.288] as mean perceived warmth was higher for conditions of high warmth (*M* = 4.51, *SD* = 1.56) than low warmth (*M* = 2.64, *SD* = 1.40). Additionally, according to our manipulation, manipulated anthropomorphism had a significant effect on perceived anthropomorphism [*F*_(1,155)_ = 5.54, *p* = 0.020, η^2^_*p*_ = 0.034] as mean perceived anthropomorphism was higher for conditions of high anthropomorphism (*M* = 2.66, *SD* = 1.26) than low anthropomorphism (*M* = 2.22, *SD* = 1.08).

#### Hypotheses Testing

Two separate two-way ANOVAs were conducted to test the assumed effects of warmth and anthropomorphism on anticipated trust (H1a, H3a) and attributed trustworthiness (H1b, H3b).

Regarding anticipated trust, the conducted two-way ANOVA showed a significant effect of manipulated warmth [*F*_(3,153)_ = 5.09, *p* = *0.0*26, η^2^_*p*_ = 0.032], but not manipulated anthropomorphism [*F*_(3,153)_ = 0.30, *p* = 0.588, η^2^_*p*_ = 0.002]. No interaction effect of manipulated warmth and manipulated anthropomorphism on anticipated trust [*F*_(3,153)_ = 2.67, *p* = 0.104, η^2^_*p*_ = 0.017] was found. Mean anticipated trust was higher for conditions of high warmth (*M* = 3.40; *SD* = 1.46) compared to low warmth (*M* = 2.90; *SD* = 1.36). Thus, H1a was supported. No moderation effect of manipulated anthropomorphism on the effect of manipulated warmth on anticipated trust was found. Thus, H3a was not supported.

Regarding attributed trustworthiness, the conducted two-way ANOVA showed a significant effect of manipulated warmth [*F*_(3,153)_ = 63.83, *p* < 0.001, η^2^_*p*_ = 0.294] but not manipulated anthropomorphism [*F*_(3,153)_ = 0.14, *p* = 0.708, η^2^_*p*_ = 0.001]. No interaction effect of manipulated warmth and manipulated anthropomorphism on attributed trustworthiness [*F*_(3,153)_ = 0.06, *p* = 0.801, η^2^_*p*_ < 0.001] was found. Mean attributed trustworthiness was higher for conditions of high warmth (*M* = 3.37; *SD* = 1.00) compared to low warmth (*M* = 2.22; *SD* = 0.79). Thus, H1a was supported. No moderation effect of manipulated anthropomorphism on the effect of manipulated warmth on attributed trustworthiness was found. Thus, H3a was not supported.

The conducted mediated regression analysis showed a positive total effect of manipulated warmth on anticipated trust (*B* = 0.50, *t* = 2.22, *p* = 0.028) and that perceived warmth significantly mediated this interrelation with a positive indirect effect (*B* = 0.51). A bootstrap 95% CI around the indirect effect did not contain zero (0.22; 0.85). The direct effect of manipulated warmth on anticipated trust became not significant (*B* = −0.01, *t* = −0.04, *p* = 0.965) after including the mediator variable, implying a complete mediation, and supporting H2a. A detailed overview of the mediated regression analysis is presented in Table 8.

**Table 8:** Mediated regression analysis testing the effect of manipulated warmth on anticipated trust mediated by perceived warmth in study 2.

					**Model**
**Predictor**	***B***	***SE***	***T***	***P***	***R^**2**^***
Model 1: X on Y					0.03
Intercept	2.90	0.16	18.37	<0.001	
Manipulated warmth	0.50	0.23	2.22	0.28	
Model 2: X on M					0.29
Intercept	2.64	0.17	15.89	<0.001	
Manipulated warmth	1.87	0.24	7.91	<0.001	
Model 3: X + M on Y					0.11
Intercept	2.18	0.25	8.87	<0.001	
Perceieved warmth	0.27	0.07	3.72	<0.001	
Manipulated warmth	−0.01	0.26	−0.04	0.965	

The conducted mediated regression analysis showed a positive total effect of manipulated warmth on attributed trustworthiness (*B* = 1.16, *t* = 0.14; *p* < 0.001) and that perceived warmth significantly mediated this interrelation with a positive indirect effect (*B* = 0.72). A bootstrap 95% CI around the indirect effect did not contain zero (0.12; 0.49). The direct effect of manipulated warmth on attributed trustworthiness remained significant (*B* = 0.43, *t* = 3.30, *p* = 0.001) after including the mediator variable, implying a partial mediation, and partially supporting H2b. A detailed overview of the mediated regression analysis is presented in Table 9.

**Table 9:** Mediated regression analysis testing the effect of manipulated warmth on attributed trustworthiness mediated by perceived warmth in study 2.

					**Model**
**Predictor**	***B***	***SE***	***T***	***P***	***R^**2**^***
Model 1: X on Y					0.30
Intercept	2.22	0.10	22.02	<0.001	
Manipulated warmth	1.16	0.14	8.05	<0.001	
Model 2: X on M					0.29
Intercept	2.64	0.17	15.89	<0.001	
Manipulated warmth	1.87	0.24	7.91	<0.001	
Model 3: X + M on Y					0.58
Intercept	1.20	0.13	9.50	<0.001	
Perceieved warmth	0.39	0.04	10.20	<0.001	
Manipulated warmth	0.43	0.13	3.30	0.001	

#### Exploratory Analyses

Parallel to Study 1, exploratory analyses were performed to detect possible interrelations between the studied constructs beyond our predefined hypotheses. Hence, we tested effects of manipulated warmth on perceived anthropomorphism as well as effects of manipulated anthropomorphism on perceived warmth. Two one-way ANOVAs showed no effect of manipulated warmth on perceived anthropomorphism [*F*_(1,155)_ = 0.61, *p* = 0.435; η^2^ = 0.004] as well as no effect of manipulated anthropomorphism on perceived warmth [*F*_(1,155)_ = 2.79, *p* = 0.097; η^2^ = 0.018].

Similar to Study 1, we conducted moderation analyses in parallel to the assumed interaction effect between robot warmth and robot anthropomorphism on trust (H3), however, this time considering the participants' subjective perceptions of robot warmth and robot anthropomorphism instead of the experimental factors as predictors of trust. Regarding anticipated trust as one trust measure, only perceived warmth showed as a significant predictor (*B* = 0.36, *t* = 2.37, *p* = 0.019), whereas perceived anthropomorphism (*B* = 0.21, *t* = 0.97, *p* = 0.334) and the interaction of perceived warmth and perceived anthropomorphism (*B* = −0.04, *t* = −0.74; *p* = 0.460) did not. Perceived anthropomorphism, therefore, did not moderate the effect of perceived warmth on anticipated trust. Regarding attributed trustworthiness as the other trust measure, perceived warmth (*B* = 0.28, *t* = 3.52, *p* < 0.001) as well as the interaction of perceived warmth and perceived anthropomorphism (*B* = 0.06, *t* = 2.17, *p* = 0.032) showed as significant predictors, whereas perceived anthropomorphism did not (*B* = −0.14, *t* = −1.30; *p* = 0.196). Perceived anthropomorphism, therefore, moderated the effect of perceived warmth on attributed trustworthiness. A detailed overview of the moderation analysis is presented in Table 10.

**Table 10:** Moderated regression analysis testing the effect of perceived warmth on attributed trustworthiness moderated by perceived anthropomorphism within study 2.

					**Model**
**Predictor**	***B***	***SE***	***T***	***P***	***R^**2**^***
Model					0.57
Intercept	1.55	0.28	5.55	<0.001	
Perceived warmth	0.28	0.08	3.52	<0.001	
Perceived anthropomorphism	−0.14	0.11	−1.30	0.196	
Perceived warmth ^*^ perceived anthropomorphism	0.06	0.03	2.17	0.032	

* *stand for interaction*.

### Discussion

The aim of Study 2 was to investigate the influence of robot warmth on trust in HRI as well as the role of robot anthropomorphism in this interrelation. In this regard, we manipulated robot warmth and robot anthropomorphism in videos, in which a robot consulted a human player in a shell game. In parallel to Study 1, based on the robot's behavior in this HRI, study participants provided two types of trust ratings, namely, attributed trustworthiness to the robot and anticipated trust toward the robot. In conformity with our hypotheses, manipulated warmth had a significant positive effect on anticipated trust as well as attributed trustworthiness and both interrelations were (partially) mediated by perceived warmth. Thus, according to our findings, robot warmth appears to be a possible determinant of trust development in HRI, supporting the transferability of warmth as a determinant of trust development in interpersonal interaction (e.g., Mayer et al., 1995; Fiske et al., 2007) to HRI. In addition, our results are compatible with previous HCI research (e.g., Kulms and Kopp, 2018), implying a positive effect of computer warmth on trust in computers.

Contrary to our hypotheses, manipulated anthropomorphism did not moderate the effect of manipulated warmth on the trust ratings. As elucidated in Study 1, a possible reason for this finding might be the restricted variance of anthropomorphism, due to its rather weak manipulation, based on the use of the same robot, with identical visual appearance in both conditions. Yet, exploratory analyses indicate that the perception of the robot as anthropomorphic may still play a role in this interrelation, when considering participants subjective perceptions of the determinants in questions. Namely, results showed that the individually perceived anthropomorphism moderated the effect of perceived warmth on attributed trustworthiness. These results indicate a further consideration of robot anthropomorphism, specifically its subjective perception, as a possibly relevant determinant of trust development in HRI, to be explored in combination with other known relevant determinants, such as warmth.

## General Discussion

The aim of our studies was to investigate whether the determinants competence and warmth, known to influence the development of interpersonal trust (e.g., Mayer et al., 1995; Fiske et al., 2007), influence trust development in HRI, and what role anthropomorphism plays in this interrelation. This was explored by two separate studies, one manipulating competence and anthropomorphism of a robot, and one manipulating warmth and anthropomorphism of a robot. Overall results imply a positive effect of robot competence (Study 1), as well as robot warmth (Study 2) on trust development in robots on an anticipatory as well as attributional level. These determinants thus seem relevant for trust development in HRI and support a transferability of essential trust dynamics from interpersonal interaction (Mayer et al., 1995; Fiske et al., 2007) to HRI.

Furthermore, considering the applied manipulations in both studies, anthropomorphic design cues in the robot neither influenced the interrelations of robot competence and trust (Study 1) nor robot warmth on trust (Study 2) on an anticipatory or attributional level. Yet, when considering participants' perception of the manipulated variables, an according effect was found; perceived anthropomorphism appeared to further influence the positive effect of perceived competence on attributed trustworthiness in Study 1 and perceived warmth on attributed trustworthiness in Study 2.

Our present results, then, contribute to research on trust development in HRI by highlighting the relevance of robot competence and robot warmth. Such results shed further light on the transferability of determinants of trust development from interpersonal interaction to HRI. Therefore, our research somewhat paves the way to understanding the complex network of factors in trust development within HRI. On a practical level, our results demonstrate how small differences in design within one single robot can come with significant differences in perceptions of the essential variables: robot competence, warmth, and anthropomorphism. Furthermore, our results offer first insights on design cues, which influence trust in robots and can thus be adjusted to foster appropriate levels of trust in HRI. Accordingly, the demonstration of high performance in a robot, e.g., by completing a task, as well as presenting the robot to have the same intentions as the user, can foster trust development. Furthermore, a perception of human likeness in a robot, e.g., based on a humanlike design, should be considered, as it might influence positive effects of perceived competence and perceived warmth of a robot on trust on an attributional level.

However, literature increasingly underlines consequences of overtrust in robotic systems. Robinette et al. (2017), for example, found that participants followed a robot's lead during an emergency even when it had performed incorrectly in previous demonstrations as well as when they were aware that the robot was acting wrongly. From an ethical perspective, it appears necessary to not only focus on design to foster trust in HRI but rather facilitate appropriate levels of trust. Although a detailed discussion in this regard would go beyond the scope of this paper, methods to foster appropriate levels of trust (e.g., Ullrich et al., 2021) should be considered in combination with the present research.

## Limitations and Future Research Directions

Some methodological limitations within our studies, as well as more general limitations of the present research paradigm, need to be considered. First, regarding our applied manipulations within both studies, a central methodological limitation is the use of videos due to the online character of the studies. Thus, participants did not experience real HRI. Additionally, the short-time demonstrations of HRI might not have formed an appropriate basis to observe a possible development of trust in the robot. Furthermore, the robot we used for our manipulations was a commercial one. Thus, we cannot exclude a possible influence of previous experiences and resulting subjective impressions regarding the robot-related variables of interest. Regarding our applied measures, a methodological limitation is the use of self-reported trust measures. In future studies actual trust behavior should be assessed to foster external validity of results.

On a conceptual level, we must reflect on the general limitations of investigating the psychological dynamics behind HRI by means of experimental studies. While the experimental manipulation of single (presumably relevant) variables, generally, provides high internal validity, one can question whether this reductionist approach is the most sensible to detect relevant influencing factors in a complex domain such as trust development in HRI. As also demonstrated in the present study, operationalizing a sensitive construct as trust development in HRI, as well as possible determinants in experimental online studies, is a rather difficult task and typically connected to many possible confounding effects. Such could be the choice of robot as well as previous experience with robots in general (e.g., Hancock et al., 2011). Additionally, the task the robot is confronted with, specifically its type and complexity, could further affect trust in the robot (e.g., Hancock et al., 2011). Furthermore, humans' intraindividual dispositions could play a role. Accordingly, many studies support an interrelation of the Big Five personality traits (John et al., 1991), conscientiousness, agreeableness, extraversion, and trust in robots (e.g., Haring et al., 2013; Rossi et al., 2018). Although our intended manipulations were successful in both studies, the systematic manipulation of the assumed determinants of trust development under study turned out rather challenging. As exploratory results in Study 1 suggest, our manipulation of robot anthropomorphism might have also had an influence on perceived competence of the robot. While this finding might hint at the rather complex interrelation of the determinants in question, in sum, we cannot be sure whether our manipulations actually captured what is at the heart of people's mental models of robots and the question of trust or distrust. In this sense, one could even question to what extent the utilization of models of interpersonal interaction is useful to explore what determines trust in robots.

Therefore, in addition to experimental studies built on models of interpersonal trust, a change of perspective to robots “as an own species” may form another source of valuable insights (see also Ullrich et al., 2020). In alignment with previous research on specifically robotic qualities that does not try to parallel but rather highlights robot's differences to humans in psychological variables (e.g., a robot's endless patience as a “superpower,” Welge and Hassenzahl, 2016; Dörrenbächer et al., 2020), future research could consider trust models that are unique to HRI. Such an alternative research approach could facilitate a more straightforward result interpretation and shed light on HRI-specific interrelations, which might have to date been overlooked, as they have not been discussed in comparable domains such as interpersonal interaction and thus need first-time exploration.

## Conclusion

Although research agrees on the importance of trust for effective HRI (e.g., Freedy et al., 2007; Hancock et al., 2011; van Pinxteren et al., 2019), robot-related determinants of trust development in HRI have barely been considered or systematically explored. Comparing trust in HRI to interpersonal trust, our results imply a certain transferability of competence and warmth as central determinants of trust development in interpersonal interaction (e.g., Mayer et al., 1995; Fiske et al., 2007) to HRI, and hint at a possible role of the subjective perception of anthropomorphism in this regard.

While our research offers a valuable contribution to insights on trust dynamics in HRI, it also comes with methodological and conceptual limitations. Future studies could further attempt to optimize systematic manipulations of the found, relevant determinants of trust development in HRI and investigate such in a common study by additionally ensuring real life interaction with a robot, also measuring trust behavior. On a conceptual level, a question arises of whether experimental studies and the general utilization of models from interpersonal interaction represent a suitable approach to explore a complex domain such as trust development in HRI. It might thus be promising for future research to surpass existing models of trust, e.g., from interpersonal interaction, and focus on innovative approaches that are unique to HRI and highlight robot-specific interrelations.

## Data Availability Statement

The raw data supporting the conclusions of this article will be made available by the authors on https://data.ub.uni-muenchen.de/.

## Ethics Statement

Ethical review and approval was not required for the study on human participants in accordance with the local legislation and institutional requirements.

## Author Contributions

LC, DU, and SD conceived and planned the study. LC, AG, TS-G, and DU carried out the study and performed data analyses. All authors discussed the results and contributed to the manuscript.

## Conflict of Interest

The authors declare that the research was conducted in the absence of any commercial or financial relationships that could be construed as a potential conflict of interest.
